# Nonmechanical Roles of Dystrophin and Associated Proteins in Exercise, Neuromuscular Junctions, and Brains

**DOI:** 10.3390/brainsci5030275

**Published:** 2015-07-29

**Authors:** Bailey Nichols, Shin’ichi Takeda, Toshifumi Yokota

**Affiliations:** 1 Department of Medical Genetics, University of Alberta Faculty of Medicine and Dentistry; 8812-112 St, Edmonton, AB T6G 2H7, Canada; E-Mail: bmiskew@ualberta.ca; 2 Department of Molecular Therapy, National Institute of Neuroscience, National Center of Neurology and Psychiatry, 4-1-1, Ogawa-higashi, Kodaira, Tokyo 187-8502, Japan; 3 Muscular Dystrophy Canada Research Chair, 8812-112 St, Edmonton, AB T6G 2H7, Canada

**Keywords:** dystrophin, dystrophin-glycoprotein complex (DGC), syntrophin, exercise, brain, tadalafil (Cialis^®^), sildenafil citrate (Viagra^®^), muscular dystrophy, neuronal nitric oxide synthase (nNOS), two-hit hypothesis (two-hit theory)

## Abstract

Dystrophin-glycoprotein complex (DGC) is an important structural unit in skeletal muscle that connects the cytoskeleton (f-actin) of a muscle fiber to the extracellular matrix (ECM). Several muscular dystrophies, such as Duchenne muscular dystrophy, Becker muscular dystrophy, congenital muscular dystrophies (dystroglycanopathies), and limb-girdle muscular dystrophies (sarcoglycanopathies), are caused by mutations in the different DGC components. Although many early studies indicated DGC plays a crucial mechanical role in maintaining the structural integrity of skeletal muscle, recent studies identified novel roles of DGC. Beyond a mechanical role, these DGC members play important signaling roles and act as a scaffold for various signaling pathways. For example, neuronal nitric oxide synthase (nNOS), which is localized at the muscle membrane by DGC members (dystrophin and syntrophins), plays an important role in the regulation of the blood flow during exercise. DGC also plays important roles at the neuromuscular junction (NMJ) and in the brain. In this review, we will focus on recently identified roles of DGC particularly in exercise and the brain.

## 1. Introduction—Dystrophin-glycoprotein Complex (DGC)

**Figure 1:**
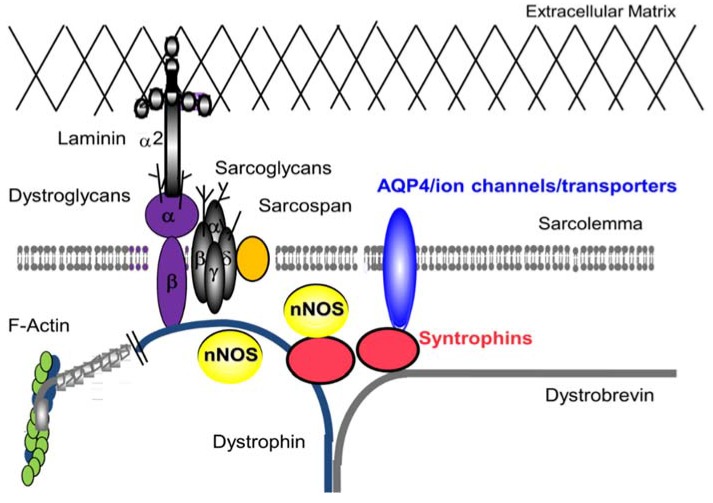
Dystrophin-Glycoprotein complex (DGC) in skeletal muscle. The DGC is a large multicomponent complex and has both mechanical stabilizing and nonmechanical signaling roles in skeletal muscle and in other organs.

Dystrophin was first identified by Hoffman and colleagues as the protein missing from X-linked Duchenne muscular dystrophy (DMD) patients in 1987 [1,2]. The *DMD* gene, the largest known human gene, was the first gene identified by the positional cloning method (also called reverse genetics) [3]. Dystrophin protein is a large cytoskeletal protein (427 KDa) which is found at the inner surface of muscle fibers. Shortly after the identification of dystrophin, Ervasti and colleagues identified a large complex of sarcolemmal (muscle membrane) proteins and glycoproteins, called the dystrophin-glycoprotein complex (DGC) (or called dystrophin-associated protein complex: DAPC) in 1990 (Figure 1) [4].

Yoshida and colleagues independently identified the same complex [5] and divided them into two sub-complex groups; they are now called the dystroglycan and the sarcoglycan complex [6]. Mutations in these complexes lead to congenital muscular dystrophies (dystroglycanopathies) and limb-girdle muscular dystrophies (LGMD; sarcoglycanopathies), respectively. Dystroglycans (α and β) and sarcoglycans (α, β, γ, δ and ε) are glycosylated on their extracellular domains. Various forms of congenital muscular dystrophies are caused by defects in the glycosylation of α-dystroglycan [7].

Two forms of dystrophin-deficient muscular dystrophies are DMD and Becker Muscular Dystrophy (BMD). DMD is the more severe form and is characterized by progressive muscle wasting. Young patients have motor delays and gait disturbances. Loss of walking ability occurs leading to required use of a wheel chair in the early teen years and the lifespan is shortened to 25–30 years. Death occurs due to respiratory or cardiac failure caused by weakened breathing and cardiac muscles [8,9,10,11]. BMD is a less severe form with similar symptoms but a slower progression rate. The severity of BMD varies from borderline DMD to almost asymptomatic cases [12]. LGMD are a group of disorders characterized by muscle weakness, fatigue, and atrophy in muscles of the shoulder and pelvic girdle (limb-girdle area). LGMD can become symptomatic in youth or adulthood [13].

The syntrophins and dystrobrevin are members of the cytoplasmic complex of dystrophin, and serve as a scaffold for signaling proteins [14,15]. Although no primary mutations in the corresponding genes have been described in human diseases, the syntrophins and dystrobrevin are decreased in DMD muscles. Therefore, these proteins are likely involved in the pathophysiology of DMD. Syntrophins function as modular adaptors that localize signaling molecules, such as neuronal nitric oxide synthase (nNOS) [16,17], water channel aquaporin-4 (AQP4) [18], ion channels [19], kinases [20], and transporters [21] at the muscle membrane in association with the DGC. The last protein of the DGC characterized is sarcospan [22]. Sarcospan has four transmembrane spanning domains and consensus phosphorylation sites for cyclic adenosine monophosphate (cAMP)-dependent protein kinase, protein kinase C and casein kinase II, though its role in skeletal muscle is still poorly understood.

When dystrophin was identified, we were far from expecting its various roles in many organs including blood flow regulation during exercise and in the central nervous system (CNS). Early studies conducted in 1980s and 90s indicated DGC plays a crucial role in providing the structural integrity of skeletal muscle [23]. More recent studies identified novel roles of DGC. Now, DGC members are thought to play significant signaling roles and act as a scaffold for various signaling pathways. Currently, the lack of nNOS and the dysregulation of the blood flow are thought to affect the DMD symptoms (two-hit hypothesis) [24]. In this review, we will discuss nonmechanical roles of DGC during exercise and in the brain, and their implications for research/translation into the clinic.

## 2. DGC—Non Mechanical Roles in Skeletal Muscle

The DGC is a multimeric and multifaceted protein complex. It appears to have both mechanical and nonmechanical roles in skeletal muscle. In this section, we will focus on the nonmechanical roles of the complex in skeletal muscle.

### 2.1. DGC and Muscle Fatigue

Muscle fatigue is defined as the inability of a skeletal muscle to maintain a certain force output [25]. The development of muscle fatigue is usually quantified as a decline in the maximal force of skeletal muscle. Recently, our group and others reported that DGC members play important roles in the blood flow regulation and the recovery from muscle fatigue [26,27]. Indeed, muscle fatigue is a frequent complaint in at least several forms of muscular dystrophies [13,28,29]. In skeletal muscle, dystrophin associates with various proteins and glycoproteins to form the DGC [30,31]. Neuronal nitric oxide synthases (nNOS) is an enzyme catalyzing the production of nitric oxide (NO) from l-arginine, and bind to a dystrophin complex member, syntrophin [32,33]. nNOS also binds to the central rod domain of dystrophin (Figure 1). NO is an important cellular signaling molecule, diffusing into the underlying smooth muscle cells in blood vessel, and causing them to relax and thus permit the surge of blood to pass through easily. The DCG acts as a connector allowing α1-syntrophin to anchor nNOS to the sarcolemma [13]. nNOS also binds to the central rod domain of dystrophin, and the sarcoglycan complex is essential for its sarcolemmal localization. The loss of nNOS from the skeletal muscle sarcolemma exacerbates the fatigue experienced after mild exercise in the mouse model [26]. A study done by Kobayashi and colleagues showed an impaired recovery from fatigue in several mouse models with mutations in dystrophin complex members including dystrophin, sarcoglycan, and nNOS. These mouse models exhibited decreased activity after the treadmill exercise. nNOS-null quadriceps skeletal muscle arteries after exercise marked the extended area of vascular narrowing, indicating that nNOS expression in skeletal muscle is required to maintain activity after mild exercise [26]. Therefore, nNOS likely plays an important role in recovery from muscle fatigue. In the wild type (WT) case, nNOS localized to the sarcolemma causes contraction induced signalling. NO synthesized by nNOS diffuses into the vascular smooth muscles and activates guanylyl cyclase. Then, it catalyzes the dephosphorylation and converts guanosine-5′-triphosphate (GTP) to cyclic guanosine monophosphate (cGMP). cGMP serves as a second messenger for relaxation of smooth muscle. In the mouse model, decreased contraction induced signalling leads to lowered cGMP and narrowed vessels [26].

**Figure 2:**
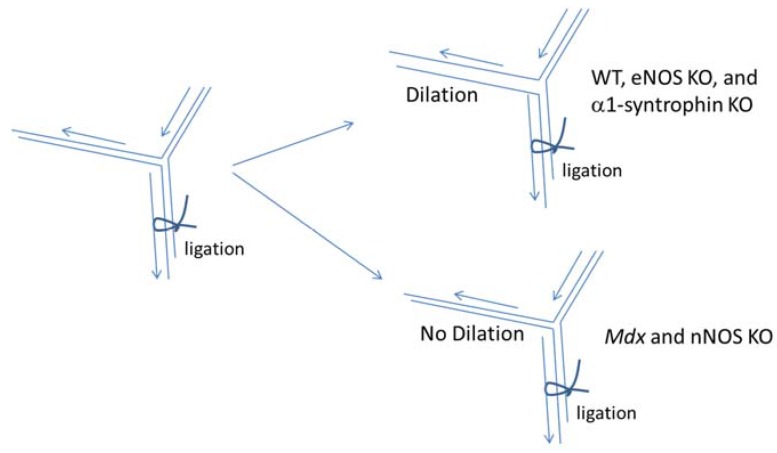
nNOS expression in skeletal muscle is the main supplier of nitric oxide in shear stress-induced vasodilation. Shear stress-induced vasodilation was significantly impaired in *mdx* mice and nNOS deficient (nNOS KO) mice compared with that of WT (C57BL/B10 or C57BL/6). Although nNOS KO mice show impaired vasodilation, eNOS deficient (eNOS KO) mice and α1-syntrophin deficient mice (α1-syntrophin KO) do not show significant differences in the dilatory ratio compared with that of WT (C57BL/6) suggesting that nNOS is the main supplier of NO for the shear stress-induced vasodilation of arterioles in skeletal muscle and that the intramuscular localization of nNOS in the skeletal muscle is not critical for shear stress-induced vasodilation.

Sato *et al*. found that vasodilation of intramuscular arterioles under shear stress in dystrophin-deficient skeletal muscle is impaired through decreased nNOS expression (Figure 2) [34]. In this experiment, they examined the *in vivo* effects of shear stress-induced dilation of mouse cremaster arterioles of wild type (WT; C57BL/10, C57BL/6), dystrophin deficient *mdx*, α1-syntrophin deficient mice, endothelial NOS (eNOS) deficient mice, and nNOS deficient mice after the ligation of the artery. In WT mice, the ligation led to the dilation of the other branch of the artery allowing a compensatory increase in blood flow.

**Figure 3:**
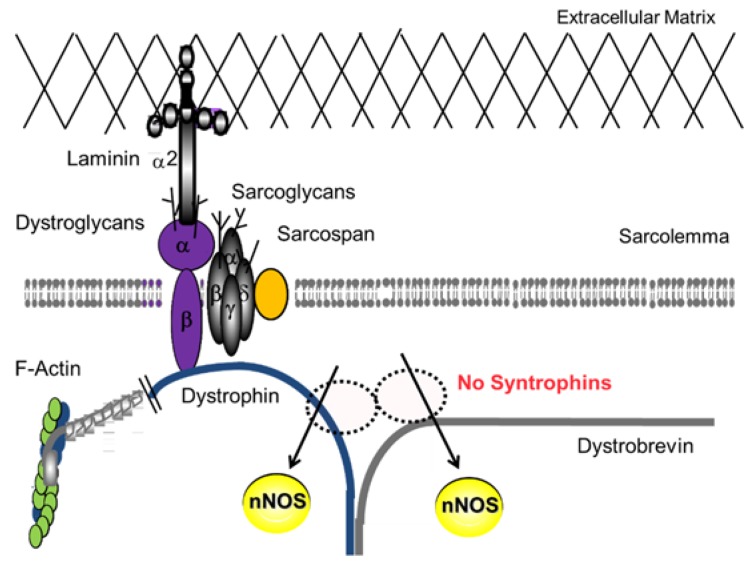
The decreased nNOS localization at the sarcolemma and the increase in cytosolic nNOS in α1-syntrophin null mice. In skeletal muscle, nNOS localization is regulated by α1-syntrophin. Despite the reduction in nNOS localized at the sarcolemma, nNOS activity is present in cytosol in the mutant mice.

The vasodilation was significantly impaired in *mdx* mice as well as nNOS deficient mice. In contrast to nNOS deficient muscles, the vasodilation under shear stress in α1-syntrophin deficient mice was normal [34]. Interestingly, α1-syntrophin deficient mice did not show significant differences in the dilation in spite of the lack of nNOS expression at the muscle membrane [34]. In α1-syntrophin deficient mice, the total expression levels of nNOS in skeletal muscle are unchanged; however, the localization of nNOS is shifted to the cytosol [17] (Figure 3), while nNOS expression levels and enzyme activity were nearly absent in DMD patients and *mdx* mice [35]. Taken together, these studies indicate nNOS is the main supplier of nitric oxide in shear stress-induced vasodilation in skeletal muscle, although the sarcolemmal localization of nNOS is not indispensable for the function. This impairment may be involved in phenotypes of DMD, sarcoglycanopathies, and dystroglycanopathies, not only in skeletal muscle but also in cardiac muscle. Clinical consequences for dystrophinopathies include muscle weakness, fatigue, muscle cramping, muscle pain (myalgia), cardiomyopathy, myoglobinuria, and in severe cases rhabdomyolysis. Corrado Angelini *et al.* in 2014 showed that decreases in nNOS localization at the sarcolemmal correlated to earlier onset, increased severity and decreases cardiac healthy in LGMD patients [13].

### 2.2. DGC and Two-Hit Hypothesis

**Figure 4:**
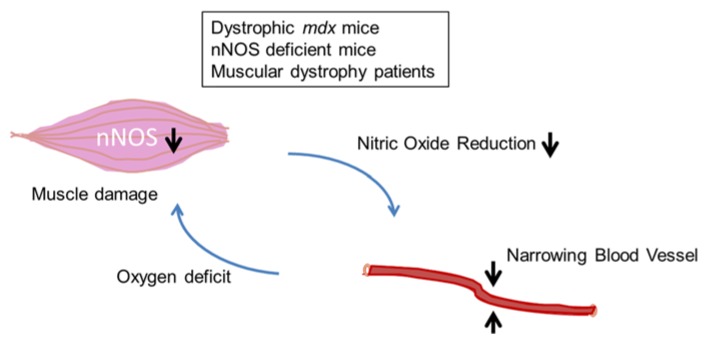
The two-hit hypothesis (two-hit theory) for myofiber damage and the effects of the functional ischemia on muscular dystrophy and animal models. The functional ischemia is caused by reduced nitric oxide (NO). The oxygen deficit leads to greater cellular susceptibility to metabolic stress and myofiber damage observed in muscular dystrophy (e.g., dystrophinopathies, and possibly sarcoglycanopathies and dystroglycanopathies).

Studies of muscular dystrophies with DGC defects suggest that one mechanism of skeletal muscle damage is the functional ischemia caused by alterations in cellular nNOS and the lack of normal dilation of blood vessels with NO reduction [36] (Figure 4). The protective action of nNOS is thought to prevent local ischemia during muscle contraction-induced increases in vasoconstriction. However, nNOS knockout mice do not develop muscular dystrophy. Thus, the loss of nNOS alone does not explain muscle degeneration. Rather, the lack of nNOS is an additive effect on these DGC defects, and these changes are called “two hit” hypothesis (or theory) of the pathogenetic mechanisms that underlie the muscular dystrophies.

**Figure 5:**
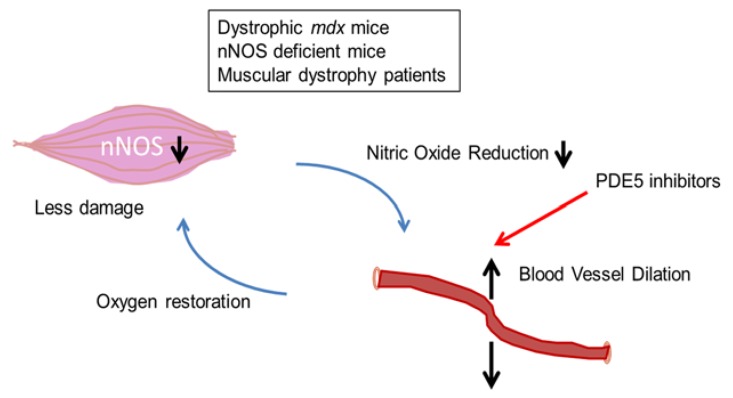
PDE5 inhibitors (e.g., Cialis and Viagra) increase the cyclic GMP levels in blood vessels that supply oxygen to skeletal muscles. PDE5 inhibitors are possibly effective for treating or preventing activity-induced fatigue and muscle damage in muscular dystrophy patients (e.g., dystrophinopathies, and possibly sarcoglycanopathies and dystroglycanopathies).

Phosphodiesterase type 5 (PDE5) inhibitors, such as tadalafil (cialis^®^) or sildenafil citrate (viagra^®^), inhibit the degradation of cGMP, increasing nNOS-mediated blood flow. Nelson *et al*. examined the effects of single oral doses of tadalafil or sildenafil on exercise-induced attenuation of reflex sympathetic vasoconstriction, a protective mechanism that promotes oxygen delivery to skeletal muscle [37] (Figure 5). PDE5 inhibition with standard clinical doses of either tadalafil or sildenafil alleviated the exercise-induced ischemia in DMD boys in a dose-dependent manner. In addition, PDE5 inhibitors normalized the exercise-induced increase in skeletal muscle blood flow. Tadalafil also alleviates muscle ischemia in patients with Becker muscular dystrophy [38]. These studies provide in-human proof of concept for PDE5 inhibition as a putative new therapeutic strategy for DMD, that is worthy of continued study.

### 2.3. Role of AQP4 in Skeletal Muscle

**Figure 6:**
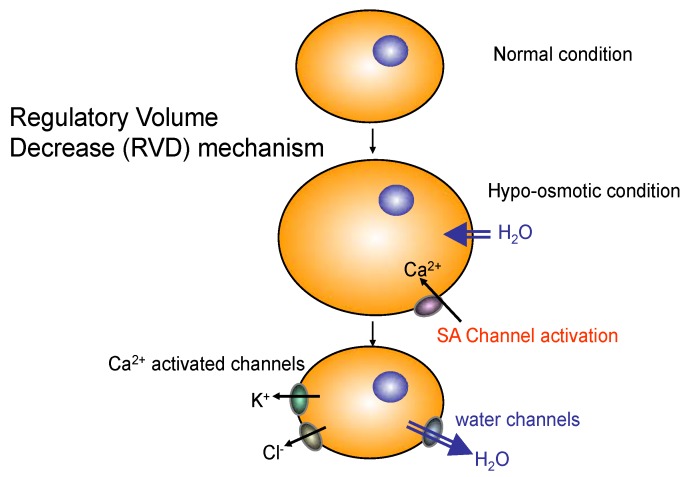
The role of water channels in cell volume regulation against osmotic shock. Acute cell volume regulation is mediated by the activation of membrane ion transporters and water channels. Regulatory volume decrease (RVD) is mediated by simultaneous activation of stress activated (SA) Ca^2+^ channels, volume-sensitive potassium and anion channels and/or electroneutral K^+^, Cl^−^ co-transporters. Volume-sensitive anion channels are permeable to Cl^−^ and organic osmolytes (e.g., amino acids). Chronic hypo-osmolarity adaptation is accomplished by decreased expression of enzymes involved in osmolyte synthesis and a decrease in organic osmolyte content.

Aquaporin-4 (AQP4) is a member of integral membrane proteins aquaporin family that conduct water through the cell membrane. Although no known muscle disease is associated with AQP4, AQP4-antibody induces astrocytic necrosis in neuromyelitis optica (NMO), which has been classified as a subtype of multiple sclerosis (MS) [39]. In skeletal muscle, AQP4 is selectively expressed in fast twitch (type II) fibers. AQP4 is also recruited by α-syntrophin, an important member of DGC, at the membrane of skeletal muscle and brain astrocyte endfeet [18]. However, the physiological functions of AQP4 in skeletal muscle are poorly understood. Aquaporins in other tissues are involved in regulatory volume increase (RVI) or regulatory volume decrease (RVD) upon osmotic shocks (Figure 6).

Increased activity of fast twitch skeletal muscle fibres results in acidosis and hyper-osmolality (by 15 mOsm) due to increased intracellular fluid lactate concentration (5–29 mM or more). Controversy exists as to whether the skeletal muscle in mammals is capable of volume regulation such as regulatory volume increase (RVI) and decrease (RVD) in response to changes in osmotic conditions. Immunofluorescence and immunoblotting experiments performed with affinity-purified antibodies revealed that only AQP4 is expressed in mouse skeletal muscle membrane among aquaporin family members [40]. Although AQP1 is also expressed in skeletal muscles, the expression was only at the intramuscular capillary endothelial cells [41]. Among 13 family members of aquaporins, AQP4 is the major water channel of the neuromuscular system; however, its physiological function in both brain and skeletal muscle is unclear [42]. α1-Syntrophin contains a post synaptic density protein (PSD95), Drosophila disc large tumor suppressor (Dlg1), and zonula occludens-1 protein (zo-1) (PDZ) domain, an acronym combining the first letters of three proteins—PSD95, Dlg1, and zo-1 [43]. The PDZ domain of α1-syntrophin is involved in the membrane localization of several key molecules, including nNOS and AQP4 [17,33,44,45]. The α1-syntrophin-null skeletal muscle was not pathogenic and showed normal contractile properties [17]. Importantly, AQP4 and nNOS*-*deficient muscle histology was not different from that of wild-type muscle either [42,46]. Nevertheless, the α1-syntrophin deficient mice exhibit many interesting characteristics, such as abnormal regeneration of skeletal muscles and neuromuscular junctions (NMJs), as well as mislocalization of molecules including AQP4, ATP-binding cassette transporter A1 (ABCA1), and nNOS [17,18,21,45].

We recently proposed that water channel AQP4 is involved in the recovery from the muscle fatigue [27]. In this study, we demonstrated that α1-syntrophin deficient skeletal muscles (isolated muscle bundles) showed impaired muscle force recovery after osmotic shock [27]. Isolated muscle bundles of fast twitch extensor digitorum longus (EDL) muscles from the mutant mouse model showed remarkably reduced force production after hypo- and hyper-osmotic shocks. In addition, the mutant EDL muscle bundles showed delayed restoration of tissue specific gravity after being exposed to hypo-osmotic conditions. These results indicate that fast twitch skeletal muscles are capable of the volume regulation (RVI and RVD) in response to changes in osmotic conditions, and it is impaired in α1-syntrophin deficient mice. However, it is not clear whether AQP4 is involved in this process or other proteins that interact with α1-syntrophin are involved. Two consecutive exercise tests with the treadmill running revealed that their running performance in the second test after short intervals was significantly lower than that of wild-type C57BL/6 mice. Since mislocalization of nNOS does not cause ischemia in α1-syntrophin deficient mice (Figure 2), the nNOS mislocalization is less likely the reason for the impaired recovery after osmotic shocks and treadmill exercise in α1-syntrophin deficient mice.

## 3. DGC Complex at the Neuromuscular Junction

**Figure 7:**
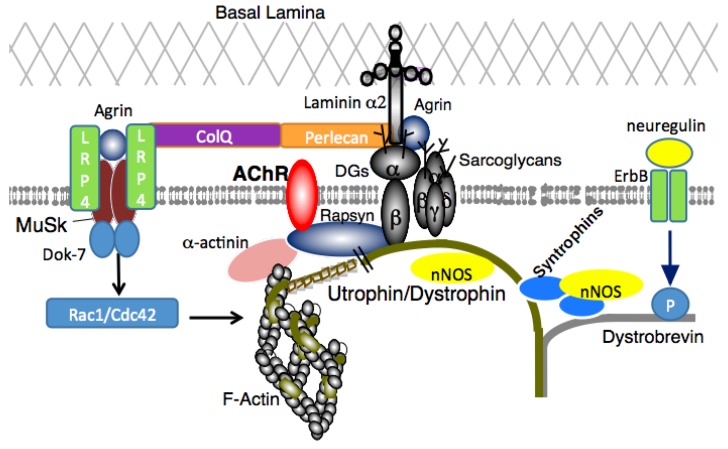
The utrophin/dystrophin protein complex and AChR. Acetylcholine Receptors **(**AChRs) are connected to the utrophin/dystrophin-associated protein complex by rapsyn, which is essential for AChR stabilization. AChR, acetylcholine receptor; ColQ, collagen Q; DG, dystroglycan; ErbB, erythroblastic leukemia viral oncogene homolog; LRP4, low-density lipoprotein receptor-related protein 4; MuSK, muscle-specific kinase; nNOS, neuronal nitric oxide synthase. This localization occurs at the neuromuscular junction.

The neuromuscular junction (NMJ) is a type of synapses that connects a motor neuron and a skeletal muscle fiber. The NMJ exhibits a high degree of subcellular specialization, and the development and maintenance of NMJs are highly complex and orchestrated by numerous proteins. DGC highly accumulates at the NMJ and at a variety of synapses in the peripheral and central nervous systems (Figure 7).

In NMJs, utrophin, a dystrophin-related cytoskeletal protein expressed in many tissues, is precisely colocalized with acetylcholine receptors (AChRs), and involved in AChR cluster formation or maintenance [47,48,49]. Postsynaptic abnormalities at the NMJs are reported in several mutant mice with defects in utrophin/dystrophin complex members, including utrophin-deficient mice, utrophin-dystrophin double deficient mice, and syntrophin deficient mice [45,50,51,52]. In addition, deficient mice of dystroglycan and α-dystrobrevin exhibit reduced AChR clustering at the NMJ [53,54,55]. Importantly, α1-syntrophin deficient mice do not develop muscle degeneration. Therefore, the impaired muscle force generation after muscle regeneration induced by cardiotoxin in the mutant mice is likely due to the abnormal formation of NMJs. Shiao and colleagues reported that defects in NMJ structure in dystrophic muscle are corrected by expression of a NOS transgene in dystrophin-deficient muscles but not in muscles lacking α1- and β1-syntrophins. Since syntrophins are involved in the membrane localization of nNOS, these results strongly indicate that nNOS at the sarcolemma promotes AChR expression and clustering at the NMJs, and contributes to normal NMJ structure.

In 2011, Schmidt *et al.* identified an important role of α-dystrobrevin, a member of DGC, in the development of NMJs [56]. They employed mice in which neuregulin (NRG) signaling to muscle was genetically or pharmacologically eliminated. The loss of neuromuscular NRG/ErbB signaling disrupted AChR anchoring at the postsynaptic sarcolemma which was caused by dephosphorylation of α-dystrobrevin1. Bartoli and colleagues identified interactions between dystroglycan and RING-H2 domain of rapsyn, a subsarcolemmal protein in skeletal muscle necessary for the NMJ formation and AChR clustering [57]. In addition, perlecan binds to DG and ColQ. ColQ interacts with perlecan/dystroglycan and the muscle specific receptor tyrosine kinase (MuSK). They are key molecules in the NMJ and involved in the clustering of acetylcholinesterase and AChRs.

Many years of research into the roles of the DGC at NMJs have revealed that the utrophin/dystrophin complex plays an important role in the maturation of AChR complex and maintenance of NMJs. The challenges include the use of these findings to identify therapeutics. The muscle protein Dok-7 is essential for activation of the receptor kinase MuSK, which governs NMJ formation, and DOK7 mutations underlie familial limb-girdle myasthenia (DOK7 myasthenia), a neuromuscular disease characterized by small NMJs. Recently, Arimura *et al.* demonstrated that intraperitoneal injections of an adeno-associated virus (AAV) vector encoding the human *DOK7* gene lead to an enlargement of NMJs and significant increases in muscle force and lifespan in a mouse model of congenital myasthenia [58]. Surprisingly, the *DOK7* gene therapy also improved the motor activity and life span of autosomal dominant Emery-Dreifuss muscular dystrophy model mice, suggesting that gene therapies aimed at enlarging the NMJ might be effective on many other neuromuscular disorders.

## 4. DGC Complex in Brain and the Cognitive Impairment in DMD

DMD patients have been shown to struggle on specific cognitive tasks, especially those tasks that required verbal intelligence [59,60,61]. The average intelligence quotient (IQ) of DMD patients is one standard deviation below the normal population [61,62]. While many patients have minor cognitive disabilities, there is great heterogeneity within the group ranging from mild to severe mental retardation [59]. Speech and reading skills may be delayed [63,64]. They struggle with both short term and long term memory, specifically visuospatial functions [65]. Decreased achievement on digit span and story recall is reported in those with DMD [64]. Comorbidity with various psychiatric disorders, such as autism spectrum disorder, anxiety and depression, and behavioral problems are common [66,67,68,69]. This section looks at the role of dystrophin in the brain, and the possible cause of mental retardation due to the mutations within the *dystrophin* gene.

### 4.1. Regions of the Brain Affected

Based on patient symptoms and mouse models, it is believed that the hippocampus, cerebellum, amygdala, and cortical regions of the brain are affected [63,70]. Dystrophin isoforms assumed to be associated with the cognitive deficits in DMD patients were found in these regions [63,71,72,73]. *Mdx* mice have poor motor coordination possibly indicating cerebellar dysfunction or a result of muscular weakening [70,74,75]. Sekiguchi *et al.* showed that *mdx* mice have abnormal functioning within the amygdala, indicated by abnormal defensive behaviors [76]. DMD patients have difficulty with identifying facial expression, also suggesting amygdala dysfunction [63,77]. *Mdx* mice show some memory deficits providing evidence for hippocampal dysfunction. Various groups showed these mice struggled with bar pressing tasks and spatial memory tasks when there were delays in original training and testing, suggesting an impairment in long-term memory[75,78,79]. Based on this, dystrophin is thought to function in memory consolidation or expression of long-term memories[63]. DMD patients have deficits in all types of memory including working, short-term and long-term memory, again suggesting some role of dystrophin in memory[59,80].

### 4.2. Dystrophin Protein Isoforms

**Figure 8:**
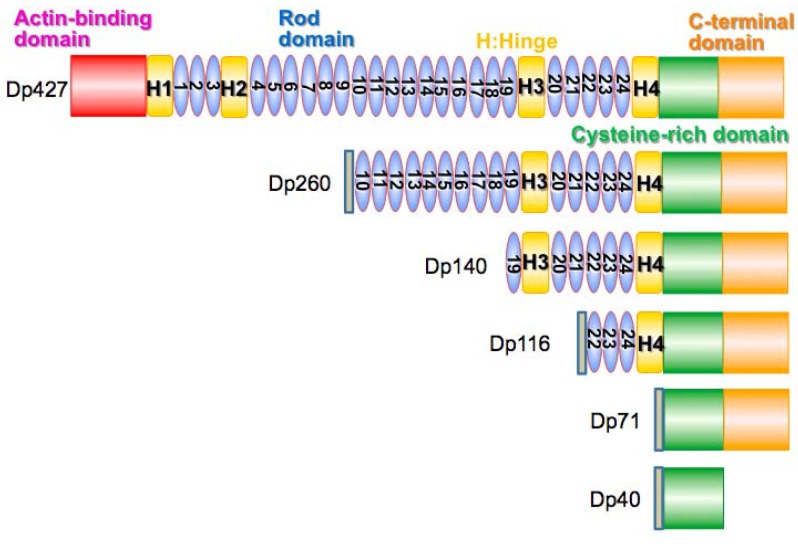
DMD/dystrophin protein (Dp) isoforms. The *DMD* (*dystrophin)* gene produces various dystrophin isoforms. The main full-length dystrophin protein is called Dp427. In skeletal muscle, DP427 has a unique N-terminal amino acid sequence, MLWWEEVEDCY, and called Dp427m (muscle) [83]. In neurons, the MLWWEEVEDCY-start sequence of Dp427m is replaced by a unique N-terminal MED amino acid sequence, and called Dp427c (cortex) [84]. In Purkinje cells in the brain, a unique N-terminal MSEVSSD amino acid sequence is expressed, and called Dp427p (Purkinje) [85]. The full-length dystrophin central rod domain contains 24 spectrin-like repeats. Dp260 is a C-terminal isoform of dystrophin expressed by an alternative promoter located in intron 29 in the *DMD* gene [86]. Dp260 is mainly expressed in retina, and contains 15 spectrin-like rod repeats in the rod domain. Dp140 is a C-terminal dystrophin isoform, mainly expressed during fetal brain development with an alternative promoter in intron 44. Dp140 contains six spectrin-like rod repeats, and the loss of Dp140 is associated with a higher risk of cognitive impairment in DMD and BMD [87]. Dp116 is an alternative C-terminal dystrophin short isoform, specific to Schwann cells in peripheral nerve [88]. Dp71 is a ubiquitous isoform of dystrophin expressed with an alternative promoter located in intron 62 [89]. Dp40 is the shortest known isoform of dystrophin expressed in hippocampal neuron. Dp40 is known to interact with various presynaptic proteins [90,91].

Location of the mutation in the *DMD* gene seems to be particularly important for determining the degree of brain dysfunction. Short C-terminal proteins, Dp71 and Dp140 have been found at high concentrations in affected brain areas within mouse models of DMD (Figure 8) [79,81]. This leads to the suggestion that they may play a significant role in the development of DMD with mental deficits. Work by Vaillend and Ungerer with *mdx3cv* mutant, which have reduced expression of dystrophin and C-terminal products including Dp71 and Dp140 due to a point mutation within intron 65, brought into question the role of these proteins causing mental impairments in DMD patients. Using the bar pressing task and the T-maze, they found that the *mdx3cv* mice had similar or fewer learning and memory deficits compared to the *mdx* mice. However, the authors admit that the methods of obtaining the mutant mice spontaneously may have caused confounding results. Thus further research was needed to decide if Dp71 and Dp140 have causal effects on the mental capacity of DMD boys [79]. Current evidence indicates mutations affecting dystrophin at three sites—Dp71, Dp140 and Dp427 are responsible for more severe forms of brain dysfunction in DMD (Figure 8) [63,82].

Dp71-null mice do not have the same muscle pathology as the *mdx* mice, but show abnormal behavior and cognitive deficits, such as poor spatial learning. Dp71-null mice were generated by the use homologous recombination techniques. Starting with SVJ mice, parts of exon one and intron one of Dp71 were exchanged with a sequence for β-gal-neomycin-resistance chimeric protein (β-geo). This prevented expression of Dp71 without affecting other dystrophin products [92,93]. Increased calcium levels in cerebellar granule cells, similar but less significant than what is seen in DMD muscle, has been observed in the mouse model brain [94]. This can cause increased vulnerability to necrosis in cells where Dp71 is absent or improperly functioning to form β-dystroglycan complexes [71]. In 2001 work by Culligan *et al.* on the *mdx3cv* mouse model showed that the Dp71 isoform disrupted anchoring of dystroglycans, which the authors believe may destabilize brain structures, such as the blood brain barrier (BBB), and signalling pathways [71]. Dp71 null mice show decreased density of excitatory synapses and increased glutamatergic transmission [63,95]. Dp71 has been detected in both neurons and supporting glial cells suggesting more than one function [63]. Dp71 may play a role in the BBB since it is expressed where glial cells interact with the vascular system [63,96]. However, there is not sufficient evidence to conclude that this causes poor plasticity and cognitive functioning in DMD patients. Dp71 functions in the aggregation of two channels—one being a water channel, AQP4. AQP4 is part of the DGC and increases vascular permeability upon damage to the BBB [97,98,99]. When Dp71 is decreased, there is a reduction in AQP4 channels [97]. The second is a potassium ion channel, Kir4.1 [63,100]. This also suggests a role in blood barrier functioning, as well as water and K^+^ regulation within glial cells. In retinal cells, Dp71 acts as an anchor for Kir4.1 and AQP4 and functions in retinal vascular permeability. To date, no evidence has been found to show deregulated vascular permeability in the brains of Dp71-null mice [63,101,102]. Dysfunction of the BBB can lead to increased edema and susceptibility to hypoxia [63,97]. Daoud *et al.* detected Dp71 with signaling and scaffolding proteins associated with glutamate receptors present at excitatory synapses using immunoprecipitation techniques [95]. Although other studies have been mixed on their support for this finding [63,103,104,105]. Dp71-null mice were shown to have deregulation in the osmoregulatory axis These findings inspired Benabdesselam *et al.* to look at the result of Dp71 absence on other DGC and nNOS expression in Dp71-null mice. They found decreases in β-DG and α1-syntrophin. This had effects on astrocyte morphology and, as previously suggested, BBB. They also found that when Dp71 was absent Dp140 expression increased [106,107]. This result is still in need of explanation. Dp71 has also been shown to function separately of DGC in cell adhesion, maintain nuclear architect and play a role in cell cycle division. Most recently, Suárez-Sánchez *et al.* showed Dp71 functions in nucleocytoplasmic shuttling [108,109,110,111,112,113]. Dp71 has many functions and seems to play a key factor in the development of mental retardation in DMD patients.

Less is known about Dp140. Based on mice models and patient studies on brain metabolites it was thought Dp140 may be related to the suspected metabolite alterations in DMD [71]. Work in 2011 showed that not only were the metabolite abnormalities less strong than initially suggested, but that Dp140 was very weakly correlated to the changes that were present [114]. Using neuroimaging, Doorenweerd *et al.* found that Dp140 negative patients had the most severe decrease in grey matter and performed most poorly on neurocognitive tests compared to controls [65]. Along with previous information that Dp140 is found to be most active during fetal brain development, this suggests Dp140 functions in cerebral development [65,72,115]. Though further work should be done to ensure that these differences are not secondarily caused by steroid treatment [65].

Dp427 has been studied in the *mdx* mouse, as this product is absent in this model [71,116]. It has been localized to cerebral Purkinje cells, cortical and hippocampal neurons and is primarily found post synaptically [71,72,73]. No abnormalities in gross brain anatomy in *mdx* mice or DMD patients has been observed, suggesting a role at the cellular level [117]. Work done in 1992 on CA1 hippocampal neurons suggests dystrophin functions at synapses within the brain [63,118]. Changes in long-term synaptic plasticity have been observed in both the cerebellum and the hippocampus. In 2004, Anderson *et al.* showed slices of *mdx* cerebellum had lower than normal long-term depression in Purkinje cells [119]. Similarly abnormalities in long-term depression were shown in *mdx* hippocampus; however, increased activity was noted here. *N*-methyl-d-aspartate **(**NMDA)-dependent receptors in *mdx* hippocampus showed increased short term and long-term potentiation. Gamma-aminobutyric acid (GABA), largely an inhibitory neurotransmitter, binds to a large group of receptors; one being the GABA_A_ receptor. The GABA_A_ receptor has a subunit, α2, co-localized with dystrophin at the inhibitory synapse. In *mdx* mice, the affected regions, amygdala, hippocampus and cerebellum, all show decreased levels of GABA_A_ receptors [63]. The cerebellum is affected at the α1 subunit. Kueh *et al.* showed an increase in extra-synaptic GABA_A_ receptor clusters. This suggests a role of dystrophin in sequestering GABA_A_ receptors [120]. Increased frequency of miniature inhibitory postsynaptic currents (mIPSCs) in CA1 was identified in the *mdx* in 2008. It received further validation by Tongo *et al.* in 2009 when parvalbumin (PV) expressing GABAergic interneurons were shown to be increased in the *mdx* mouse. After further looking into these results, the hypothesis is that increased pre-synaptic activity is the cause [63,120,121,122,123]. A study in 2009 that detected an increased number of inhibitory synapses may also explain this [124]. Dystrophin seems to play a role later on in development of GABA_A_ receptors at the synapse [63,125,126].

### 4.3. Potential Treatments

Sekiguchi *et al.* showed that the use of antisense morpholino oligonucleotides in *mdx* mice decreased abnormal cognitive behaviors [76]. Antisense morpholinos were designed to skip exon 23 of the *DMD* gene. This results in a shortened but functional dystrophin protein [127,128,129]. As discussed earlier, Sekiguchi *et al.* found that *mdx* mice showed an abnormal freezing response in a variety of behavioral tests [63,76]. Antisense morpholino was given intracerebroventricular to *mdx* mice. PCR evidence showed truncated dystrophin bands indicating that exon skipping occurred in treated mice. After administration of antisense morpholinos *mdx* mice were put through behavioral tests and decreased defensive freezing was observed. Antisense morpholino therapy could potentially be used to improve the behavioral problems in DMD patients [76]. Work by Tamma *et al.* in 2013 showed that α-methyl-prednisolone (PDN), a glucocorticoid, functions to improve the abnormalities in the BBB of *mdx* mice. *Mdx* mice were given intravascular injections of PDN; increased blood brain barrier markers were observed. Decreased AQP4 phosphorylation and β-DG was also observed. They suspect PDN targets epithelial and glial cells acting to rehabilitate the dysfunctional interactions between the dystrophin associated proteins (DAP) and extracellular matrix seen in *mdx* mice. PDN is thought to cause these actions by inactivating protein kinases. This information could help to develop new therapies for the cognitive deficits associated with DMD in the future [97]. Further research is needed to find a sufficient treatment for DMD patients with cognitive deficits, but current work has indicated that there may be potential targets for improving functioning.

## 5. Conclusions

Dystrophin was first studied as a protein connected to muscular dystrophy [1]. Further work revealed it was part of a large complex known as the DGC. Included in this complex are dystrophin, syntrophins, dystrobrevin, AQP4, nNOS, sarcoglycans (α, β, γ, δ, ε), dystroglycans (α and β), and sarcospan [4]. It was originally understood to function in structural support of muscle but has since been discovered to have many other functions. This includes roles within the brain, acting as scaffolding for signal pathways, roles in the NMJ, blood flow regulation and muscle fatigue. During exercise, mice with dystrophin mutations showed increased fatigue—this is believed to be the result of the two hit hypothesis of ischemia. nNOS loss from DAPC and impaired vasodilation result in an ischemic state in muscles after exercise [24]. Treatment with PDE5 inhibitors decreases the fatigue effects seen in muscular dystrophy [38]. The function of AQP4 in skeletal muscle is unclear, but a study indicates it functions in muscle fatigue recovery [27].Normal fast-twitch muscles are able to regulate the myofiber volume after osmotic shock to improve fatigue recovery. These functions are lost in muscular dystrophy, weakening muscle recovery. Components of the DGC are also important at the NMJ—nNOS at the sarcolemma aids in expression of AChR and recruitment of NMJs [45,50,51,52]. There has been much investigation into the role of dystrophin in the brain because a large proportion of DMD patients have cognitive and behavioral impairments. Evidence points to four affected regions within the brain—the hippocampus, cortical regions, cerebellum, and amygdala. While more investigation is required, three dystrophins, Dp71, Dp140 and Dp427, seem to be connected to the mental deficits associated with DMD. Dp71 and Dp427 are well studied and have multiple functions. Importantly, they seem to be involved in the BBB and synaptic functions [63]. Dp140 is less studied but appears to act in cerebral development. While there is no sufficient treatment for these problems faced by patients, work with antisense morpholinos and PDN provide potential future treatment options.

Since the late 80s, much work has been done on dystrophin. Knowledge on its function and multiple roles has grown substantially in that time. Its role in muscle stabilization is best understood, but new work is expanding on its novel functions. This new information has helped to better understand the varying phenotypes seen in muscular dystrophy. While great progress has been made, more work is needed to draw more precise conclusions, especially in relation to dystrophin’s role within the brain. Steroid treatment is currently the standard for treating muscular dystrophy, but the efficacy is not great. Growing knowledge in the field may lead to the development of new treatment options in the future. There are also promising treatments such as the use of morpholinos currently being tested, that, in time, could prove to be a better treatment for muscular dystrophy.
